# Emergence of Community-Associated Methicillin-Resistant *Staphylococcus aureus* Associated with Pediatric Infection in Cambodia

**DOI:** 10.1371/journal.pone.0006630

**Published:** 2009-08-13

**Authors:** Kheng Chheng, Sarah Tarquinio, Vanaporn Wuthiekanun, Lina Sin, Janjira Thaipadungpanit, Premjit Amornchai, Ngoun Chanpheaktra, Sarinna Tumapa, Hor Putchhat, Nicholas P. J. Day, Sharon J. Peacock

**Affiliations:** 1 Angkor Hospital for Children, Siem Reap, Kingdom of Cambodia; 2 Mahidol-Oxford Tropical Medicine Research Unit, Mahidol University, Bangkok, Thailand; 3 Centre for Clinical Vaccinology and Tropical Medicine, Nuffield Department of Clinical Medicine, University of Oxford, Oxford, United Kingdom; 4 Department of Medicine, University of Cambridge, Addenbrooke's Hospital, Cambridge, United Kingdom; Columbia University, United States of America

## Abstract

**Background:**

The incidence of community-associated methicillin-resistant *Staphylococcus aureus* (CA-MRSA) infection is rising in the developed world but appears to be rare in developing countries. One explanation for this difference is that resource poor countries lack the diagnostic microbiology facilities necessary to detect the presence of CA-MRSA carriage and infection.

**Methodology and Principal Findings:**

We developed diagnostic microbiology capabilities at the Angkor Hospital for Children, Siem Reap, western Cambodia in January 2006 and in the same month identified a child with severe community-acquired impetigo caused by CA-MRSA. A study was undertaken to identify and describe additional cases presenting between January 2006 and December 2007. Bacterial isolates underwent molecular characterization using multilocus sequence typing, staphylococcal cassette chromosome *mec* (SCC*mec*) typing, and PCR for the presence of the genes encoding Panton-Valentine Leukocidin (PVL). Seventeen children were identified with CA-MRSA infection, of which 11 had skin and soft tissue infection and 6 had invasive disease. The majority of cases were unrelated in time or place. Molecular characterization identified two independent MRSA clones; fifteen isolates were sequence type (ST) 834, SCC*mec* type IV, PVL gene-negative, and two isolates were ST 121, SCC*mec* type V, PVL gene-positive.

**Conclusions:**

This represents the first ever report of MRSA in Cambodia, spread of which would pose a significant threat to public health. The finding that cases were mostly unrelated in time or place suggests that these were sporadic infections in persons who were CA-MRSA carriers or contacts of carriers, rather than arising in the context of an outbreak.

## Introduction

Infections caused by methicillin-resistant *Staphylococus aureus* (MRSA) are a major scourge of modern medical care in the developed world [1]. For many years after its emergence, MRSA was associated with carriage or infection in the hospital setting where strains flourished due to the selection pressure of antibiotics but failed to become established in the community setting. A changing pattern of disease epidemiology was signalled by reports from the United States in the late 1990's of MRSA infection in otherwise healthy children who lacked exposure to an institutional health care setting or other risk factors typically associated with MRSA colonization [2], [3]. Community-associated (CA)-MRSA has since become disseminated across much of the developed world [4]–[13], and is a leading cause of bacterial infection in otherwise healthy persons in the United States where it causes the majority of all skin and soft tissue infections in patients presenting to emergency departments [14], [15]. Most CA-MRSA infections are relatively minor, but more serious manifestations include necrotizing fasciitis, pyomyositis, osteoarticular infections, and community-acquired pneumonia including severe and often fatal necrotizing pneumonia.

Characterisation of CA-MRSA isolated from a wide geographic distribution has indicated that these strains are often relatively susceptible to antimicrobials compared with their hospital-associated MRSA counterparts. Most strains carry a small variant of the methicillin-resistance cassette (SCC*mec* type IV or less often type V) [4], [7], [10]–[13], [16], and are frequently although not universally positive for the genes encoding Panton-Valentine leukocidin (PVL) [7], [13], a two-component leukolytic toxin associated with skin and soft tissue infections and more severe infections such as necrotizing pneumonia [17], [18]. Genetic comparison of a collection of CA-MRSA using multilocus sequence typing (MLST) has indicated that strains with common features have arisen in geographically dispersed *S. aureus* strains with unrelated genetic backgrounds, indicative of multiple independent clonal origins [7].

Resource-restricted Asia has largely been spared from CA-MRSA to date, despite the ready availability of over-the-counter antibiotics and frequent self-medication. The dissemination of CA-MRSA into rural Asia would represent a major threat to health. Diagnostic microbiology is often lacking and so MRSA would go unrecognised, health care including access to expensive antibiotics is restricted, and a large proportion of health care is provided by traditional healers or untrained personnel working in local dispensaries. Here, we report the identification of two independent clones of CA-MRSA associated with mild and severe infections in children presenting to the Angkor Hospital for Children (AHC) in Siem Reap, Cambodia. Infections appeared to be sporadic rather than related to an outbreak, suggesting that they were associated with endemic carriage of the causative strains in the community.

## Methods

### Ethics statement

The study protocol was reviewed and approved by the Ethical Review Board of the Angkor Hospital for Children. The Ethical Review Board deemed consent unnecessary for this retrospective study.

### Setting and Patients

The study was conducted at the AHC, an NGO-funded teaching hospital in Siem Reap, situated in the province of Siem Reap, northwest Cambodia. This city has a population of 140,000 people and is the fastest growing city in the country, a result of tourism to the nearby Angkor Wat temples. The AHC provides free outpatient, inpatient, emergency, surgical, medical, ophthalmological and dental care, and maintains 50 inpatient beds spread across high, medium and low intensity care areas. The outpatient department sees an average of approximately 400 children each day from an unrestricted catchment area, the majority of who live in three neighbouring provinces (Siem Reap, Battambang and Banteay Meanchey). Cambodia is a highly impoverished country, as reflected by recent socio-economic indicators. For example, in 2006 the mortality rate for children under 5 was 93 per 1,000 live births, the prevalence of malnutrition in the same age group was 28%, and life expectancy at birth was 59 years [19].

Laboratory-based surveillance was conducted between January 2006 and December 2007 to identify children presenting to in- or outpatient departments with one or more microbiological samples positive for MRSA. A retrospective review of case records was performed at the end of this period to collect information on age, gender, area of residence, whether admitted, diagnosis at presentation, site of *S. aureus* infection, antimicrobial therapy (type, route and duration), infection associated procedures, duration of hospital stay and outcome at hospital discharge. Sites of *S. aureus* infection were established based on a record of history and examination findings in the medical notes, together with investigation reports and operation notes. CA-MRSA was defined as an MRSA isolate recovered from a patient with *S. aureus* infection that developed in the community or within 48 hours of hospital admission, in the absence of established risk factors for MRSA infection [4], [6], [9].

### Microbiological methods

*S. aureus* was identified based on the catalase, coagulase and a commercial slide agglutination test (Staphaurex Plus, Oxoid). Susceptibilities to penicillin, oxacillin, erythromycin, trimethoprim-sulfamethoxazole, gentamicin and ciprofloxacin were tested by the hospital laboratory at the time of strain isolation using the disk diffusion method [20], and reported to treating physicians. Isolates were stored in TSB with 20% glycerol at −80°C. An extended antimicrobial susceptibility profile was later determined using the disk diffusion method to establish susceptibilities to other antimicrobial agents with potential activity against the clinical isolates. This panel was cefoxitin, chloramphenicol, ciprofloxacin, clindamycin, erythromycin, fusidic acid, gentamicin, mupirocin, netilmicin, penicillin, rifampicin, trimethoprim-sulfamethoxazole, tetracycline and vancomycin. Isolates that were resistant to cefoxitin by disk diffusion were evaluated by oxacillin and vancomycin E-tests (AB Biodisk). Inducible clindamycin resistance was determined as described previously [21].

### Molecular characterization of *S. aureus*

Genomic DNA was extracted from a 1 ml overnight culture of *S. aureus* with an optical density of 1.0 at 600 nm using the High Pure PCR Template Preparation Kit (Roche Applied Science, Germany), with an additional step of incubation at 37°C for 30 minutes with 0.5 µl of lysostaphin solution (10 mg/ml, Sigma, USA) prior to cell lysis. SCC*mec* type assignment was performed using a multiplex PCR method, as described previously [22]. Presence of the genes encoding PVL (*lukF-PV* and *lukS-PV*) was determined by PCR as described by Lina et al. [18]. MLST was performed as described by Enright et al. [23]. Sequence type (ST) assignment was based on the sequence of the alleles at each locus of seven housekeeping genes using the MLST database (www.mlst.net). STs were assigned to their clonal complex using the program eBURST (http://eburst.mlst.net) and the entire MLST database.

## Results

In December 2005, a 9 month old child presented to the outpatient department with impetigo of the outer ear and acute otitis media. The child was previously fit and well and had not been seen previously at the AHC. Culture facilities were not available, a Gram-stain of pus obtained from the skin showed Gram-positive cocci in clusters consistent with staphylococci and the child was sent home with oral cloxacillin. The child was seen again the following month with severe impetigo and acute otitis media. Diagnostic microbiology capabilities became available at the AHC in January 2006 and a swab taken from the impetigo grew MRSA. The child was admitted and made a rapid clinical response to clindamycin and gentamicin plus topical fusidic acid.

An observational study between January 2006 and December 2007 identified a further 16 children with infections caused by MRSA. The age of the 17 children ranged from 8 months to 14 years 6 months (median 4 years 8 months), 7 (39%) of who were boys. Eleven children had skin and soft tissue infection, and 6 children had invasive disease (positive blood culture and no other focus, 2 cases; osteomyelitis (one case complicated by pyomyositis), 2 cases; septic arthritis, 1 case; and pneumonia and empyema, 1 case).

Five children were treated in the outpatient department alone, and 12 were admitted to the AHC for a median duration of 13 days (range 2 to 25 days). Surgical procedures were performed on 7 children, including incision and drainage (n = 3), osteotomy for ostemyelitis (n = 2), joint washout for septic arthritis (n = 1), and chest drain insertion for empyema (n = 1). All 17 children were initially treated with first-line empiric therapy according to their admission diagnosis, with cloxacillin being the most common (n = 8). Therapy was modified thereafter based on the hospital antimicrobial susceptibility results, including ciprofloxacin in 11 cases. Extended antimicrobial susceptibility profiles defined at the end of the study are shown in Table 1.

**Table 1:** Summary data for antimicrobial susceptibility profiles of 17 CA-MRSA isolates.

Antimicrobial agent	Number of isolates resistant (%)
Chloramphenicol	2 (12%)
Ciprofloxacin	1 (6%)
Clindamycin	13 (77%)
Erythromycin	13 (77%)
Fusidic acid	0
Gentamicin	1 (6%)
Mupirocin	0
Netilmicin	0
Penicillin	17 (100%)
Rifampicin	15 (88%)
Tetracycline	16 (94%)
Trimethoprim-sulfamethoxazole	15 (88%)
Vancomycin	0

All children admitted to hospital survived to discharge. One child with MRSA-infected eczema re-presented 7 months later with rapidly progressive pneumonia and sepsis that led to death on day 2 of admission. MRSA was not isolated on this admission and the bacterial pathogen responsible was not identified. Cure was recorded for 14 of the 16 remaining children, while 2 children treated in outpatients were lost to follow up before culture results were available and outcome was unknown.

The source of MRSA acquisition was considered. Most children (16/17, 94%) had MRSA isolated from cultures taken within 48 hours of presentation and had no known risk factors for nosocomially acquired MRSA colonisation or infection. The remaining case was admitted with severe impetigo and was initially treated empirically with cloxacillin. A skin swab taken for the first time on day 6 because of failure to respond to antimicrobial therapy was positive for MRSA. There was an overlap of 3 days between this patient and a second inpatient who was receiving vancomycin for MRSA bacteremia in the absence of a known focus and who was blood culture negative on the day that the child with impetigo was admitted. The two isolates were the same genotype (sequence type (ST) 834, see below) but had variable susceptibility to erythromycin and clindamycin. We conclude that 16 cases were definite community-associated infections, and that the remaining case was most likely to have been community-associated.

**Figure 1:**
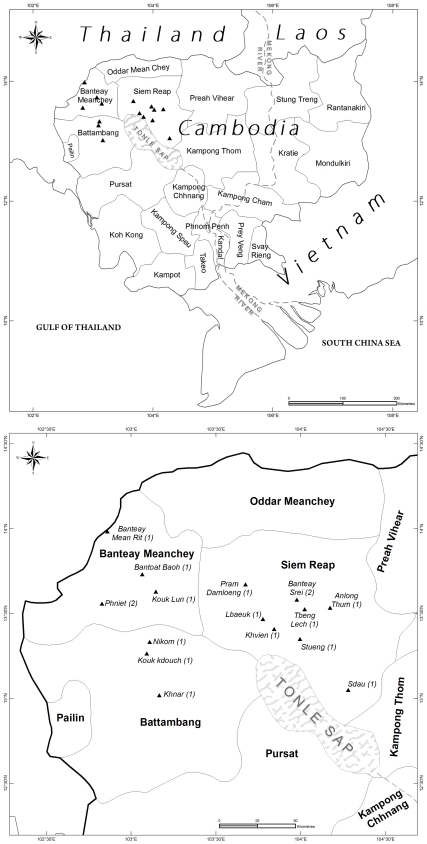
Map of Cambodia showing location of residence for 17 children with CA-MRSA infection presenting to the Angkor Hospital for Children in Siem Reap (A). Zoomed image of the three affected provinces. Numbers in brackets denote the number of children affected in each village (B).

Place of residence and timing of infection were examined to assess whether the 17 cases represented sporadic infection or whether they were related to one or more community-based outbreaks. Thirteen children resided in different villages scattered across 3 neighbouring provinces (Figure 1) and were very unlikely to have had access to any mode of transport or the opportunity to meet. Two different pairs of unrelated children lived in the same village. The first pair from a village in Banteay Meanchey province presented 23 days apart, and the second pair from a village in Siem Reap province presented 120 days apart. Each pair of children was unrelated and did not live at the same address, but it is not known whether they came into contact with each other.

Molecular characterisation of the 17 MRSA isolates demonstrated that 15 patients were infected with a strain that was designated as ST 834, SCC*mec* IV, PVL gene-negative. ST 834 has been isolated previously on three occasions during 2006 in Western Australia where it is considered to represent CA-MRSA and is colloquially known as WA MRSA-41 (personal communication, Geoff Coombs, Royal Perth Hospital). ST 834 belongs to clonal complex (CC) 9 and is a single locus variant of MRSA ST 671, which was isolated previously in Queensland in 2004. The remaining two patients were infected by isolates that were designated as ST 121, SCC*mec* type V, PVL gene-positive. The two patients infected with ST 121 lived approximately 100 km apart and were unrelated. ST 121 is the predicted founder of CC 121. ST 834 and ST 121 represent CA-MRSA in novel genetic backgrounds, since none of the previously described CA-MRSA clones belong to either of these clonal complexes. Methicillin-susceptible *S. aureus* (MSSA) ST 121 has been identified previously in the UK and Thailand, and MRSA ST 121 has been isolated in China. MLST performed by us of twenty unselected MSSA strains isolated at the AHC over the same period demonstrated that ST 121 was the largest clone and represented 12 (60%) of those tested (data not otherwise shown). The antimicrobial susceptibility pattern of MSSA ST 121 and MRSA ST 121 were comparable (data not shown), and were less antibiotic-resistant than ST 834, being susceptible to trimethoprim-sulphamethoxazole, ciprofloxacin and rifampicin against which ST 834 was resistant.

## Discussion

Published reports of CA-MRSA isolation in resource-restricted Asia are rare. An outbreak of vaccination-associated infection has been reported from Ho Chi Minh city, Vietnam caused by an isolate of CA-MRSA carried by a healthcare worker that was defined as ST 59, the cause of endemic CA-MRSA infection in Taiwan [24]. Nine clinical isolates of CA-MRSA carrying SCC*mec* type IV associated with skin and soft tissue infections have been identified in Malaysia [25]. Three MRSA were isolated from patients after 72 hours of hospitalization in a large teaching hospital in Bangkok, Thailand but the authors noted that these probably represented HA-MRSA infections [26]. A study published in 2002 of surveillance for MRSA in Battambang situated in the province adjacent to, and west of Siem Reap province reported that no MRSA was detected [27]. Our study is the first to describe MRSA of any kind in Cambodia. Cases were drawn from three different northwest Cambodian provinces, suggesting that considerable dissemination of CA-MRSA has already occurred. These provinces form the major patient catchment area for the hospital, and it is possible that CA-MRSA is present across a more extensive region of Cambodia. The majority of health care facilities across the country lack the necessary microbiology facilities to culture clinical specimens, and there is little or no awareness amongst health care workers of the possibility of MRSA.

The evidence that the MRSA isolated during this study was CA-MRSA was compelling and supported by both clinical and laboratory data. The CA-MRSA positive culture was taken within 48 hours of admission in 16 of the 17 cases from children with clinical features of *S. aureus* infection. Thirteen cases appeared to be sporadic, while two pairs of children resided in each of two villages although contact or a shared environment could not be confirmed. We conclude that most infections were indicative of endemic carriage in the community and represented discrete episodes of infection, rather than one or more outbreaks. The criteria used in the developed world that patients should have no history of recent healthcare contact may be less relevant in our setting where HA-MRSA has not been reported, but there was no evidence that CA-MRSA was acquired within a single or small number of medical facilities where *S. aureus* could spread between individuals. The molecular data on SCC*mec* type was also consistent with a community origin for these strains.

Alterations in prescribing practice has occurred over time in areas where CA-MRSA infection has become endemic, with a decrease in the use of β-lactam antibiotics as initial empirical therapy for skin and soft tissue infections and an increased use of a range of previously second-line antimicrobial agents [28], [29]. The CA-MRSA identified during this study had a high rate of resistance to several of the oral antibiotics commonly used to treat mild, community-acquired *S. aureus* infection. CA-MRSA is currently responsible for a small proportion of *S. aureus* infections overall in our setting, with isolation of MSSA from 289 clinical samples sent to the diagnostic laboratory over the period of this study (unpublished data). In view of this, we recommend that cloxacillin should continue to be used as first-line empiric therapy for suspected *S. aureus* infection. However, information regarding the possibility of CA-MRSA infection should be disseminated to healthcare workers who should consider this possibility in patients who deteriorate on appropriate therapy for MSSA infection. Vancomycin is available through donation at the AHC but is not available in government-funded hospitals in Cambodia. Possible alternatives that are available in Cambodia include the fluoroquinolones and chloramphenicol.

The clinical presentation of children presenting to our hospital with CA-MRSA infection represents a comparable spectrum to that described elsewhere, with a predominance of skin and soft tissue infections. The common circulating strains of CA-MRSA in the United States are PVL gene-positive, and an association has been made between the presence of PVL and skin and soft tissue infection. This association was absent in our setting, since most infections were caused by ST 834 which is PVL gene-negative. The two infections caused by PVL gene-positive ST 121 were osteomyelitis and soft tissue abscess, respectively.

With increasing rates of MRSA across the world, and with increasing contact between Cambodian communities and foreigners as a result of post-war humanitarian aid and more recently tourism, there is a strong possibility that Cambodia will be exposed to MRSA carriers from the developed world. However, it is not clear whether the two clones of CA-MRSA identified here arose in Cambodia or whether they arose elsewhere and were introduced by a carrier. The finding that MSSA ST 121 is the most common clone in our setting might suggest that CA-MRSA ST 121 arose locally, although MSSA ST 121 is also common in nearby countries including Thailand and China, and MRSA ST 121 has been described in China. Similarly, ST 834 has been isolated in Western Australia. The earlier time point for isolation of ST 834 from Cambodia is very weak evidence for the possibility that it arose there.

In conclusion, this study has identified CA-MRSA in three neighbouring provinces in the highly resource-restricted setting of Cambodia. Diagnostic microbiology is not available across most of the country, but we hypothesize that CA-MRSA may have become widely disseminated. This potentially major threat to public health requires urgent investigation. Studies are underway to define the prevalence of CA-MRSA carriage in the community and to track the rate of CA-MRSA cases presenting to our hospital over time.
